# Department of the Arts and Sciences

**Published:** 1869-11

**Authors:** 


### DEPARTMENT

OF THE

### ARTS AND SCIENCES.

### TO THE PATRONS OF THE JOURNAL.

HAVING increased the number of our pages for the reception of literary and scientific matter, we propose still further to enhance the popularity of the JOURNAL by presenting to each subscriber a beautiful picture which shall be an ornament upon the walls and a pleasant remembrance of us.

At the commencement of the new volume, in January, 1870, each subscriber is requested to inform us which one of the pictures is selected from the following list:

THE RESURRECTION OF CHRIST, 24 x 32. After Raphael Urbin. Published in London in 1735; a splendid line engraving in wonderful preservation

MORNING. After J. Both. Size 24 x 30

EVENING. do do

Two splendid companion landscape line engravings of great beauty. Published in London in 17—, from the original pictures in the London National Gallery.

THE GREAT ANNUAL SACRIFICE AT THE TEMPLE OF APOLLO IN THE ISLAND OF DELOS. By Claude Lorraine. Size 24 x 30. Published in London in 1764. This temple stood at the entrance of a city built entirely of granite and marble by Brisichton, son of Cecrops, first king of Athens, and was afterwards enriched by the Greeks to the admiration of all antiquity.

A MARRIAGE FESTIVAL. By Claude Lorraine. Size 24 x 30. After original in London National Gallery.

This elegant work of art is companion to the last above named and of high value.

LANDSCAPE WITH CATTLE, &c. After original of Berghem. Size 24 x 30. Published in London, 1752. Very fine.

GEN. McCLELLAN ON HIS WAR HORSE. Very fine. 24 x 30.

PICTORIAL HISTORY OF OLD TESTAMENT. 22 x 28.

PICTORIAL HISTORY OF NEW TESTAMENT. 22 x 28.

SIR WALTER SCOTT IN HIS STUDY AT ABBOTTSFORD. 22 x 28.

SIGNING THE DEATH WARRANT OF LADY JANE GRAY. Very fine. Sheet 34 x 30.

CAPTURE OF MAJOR ANDRE. Very fine. Sheet 24 x 30.

SIR WALTER RALEIGH PARTING WITH HIS WIFE. Very fine. Sheet 24 x 30.

THE JOLLY FLAT BOATMAN. Sheet 24 x 30.

THE TRAPPER'S LAST SHOT. Very fine. Sheet 24 x 30.

SPARKING. A beautiful engraving. 22 x 28.

PARTING. A Highland Scene. 22 x 28.

ANNE PAGE, SLENDER AND SHALLOW. Very fine. 24 x 30.

### CABINET CHROMOS.

*Mounted on Heavy Card Board, and Ready for Framing.*

| | | | |
|---|---|---|---|
| BROKEN PLATE | 6½ x 8½. | THE RIVALS. | 6½ x 8½. |
| HELPING GRANDMA | " | THE FAVORITES. | " |
| RUSTIC BREAKFAST. | 8½ x 10¾. | FIRST GRIEF. | 6½ x 8½. |
| FEEDING BABY. | " | BROTHER'S TOILET. | " |
| TERRIER AND PUPPIES. | 5½ x 6¾. | FEEDING CHICKENS. | " |
| HEN AND CHICKENS. | " | Do PARROT. | " |
| DOG AND CAT. | " | Do SQUIRREL. | " |
| PILLAGE. | " | Do GOAT. | " |
| DEFENCE. | " | PAINTING. | 8½ x 10¾. |
| COMBAT. | " | MUSIC. | " |

To clubs of five subscribers will be added a choice of one engraving from the following list:

THE COTTER'S SATURDAY NIGHT.—One of the most exquisite parlor and fireside pictures ever issued in this country is the "Cotter's Saturday Night, from the celebrated painting by Thomas Faed, and engraved by Lemon of London, in *pure line.* It represents the cotter's home at evening time, when the cares of the day are past. It should find a place upon the wall of every fireside.

MOUNT VERNON IN THE OLDEN TIME; OR, WASHINGTON AT THIRTY YEARS OF AGE. Fine 24 x 30.

WYOMING, JUNE 30, 1778. A thrilling scene of the memorable massacre. 24 x 30.

SIGNING THE COMPACT IN THE CABIN OF THE MAYFLOWER. Fine steel. 30 x 38.

THE VILLAGE BLACKSMITH.—Herring's noble picture of "The Village Blacksmith," engraved for the Cosmopolitan Art Association by the late lamented George Patterson, one of the best of modern engravers.

FALSTAFF MUSTERING HIS RECRUITS.—The artist has chosen the scene in Shallow's office, where the "Recruits" are called in to "pass muster" before Falstaff. This exquisite and memorable scene, after vast labor and expense, was engraved for the subscribers to the Cosmopolitan Art Association, as the subscription plate for the seventh year. 25 x 30 inches.

DEPARTURE OF PILGRIM FATHERS. ...

LANDING OF PILGRIM FATHERS.

SHAKSPEARE AND HIS FRIENDS.

The foregoing list comprises a choice selection of oil chromos and rare old engravings, the original cost of which ranged from two to ten dollars each.

By the co-operation of the publisher of these pictures, we are enabled to offer our patrons this extraordinary opportunity to provide themselves with a popular journal and obtain a picture each year worth double the amount of the subscription.

---

BY contract with publishers we can furnish beautiful pictures or engravings which have been sold for $2 and $10. Every subscriber or club is entitled to one.

